# Functional Characterization of Lycopene β- and ε-Cyclases from a Lutein-Enriched Green Microalga *Chlorella sorokiniana* FZU60

**DOI:** 10.3390/md21070418

**Published:** 2023-07-23

**Authors:** Hong Fang, Junjie Liu, Ruijuan Ma, Yiping Zou, Shih-Hsin Ho, Jianfeng Chen, Youping Xie

**Affiliations:** 1 Marine Biological Manufacturing Center of Fuzhou Institute of Oceanography, Fuzhou University, Fuzhou 350108, China; 18756987533@163.com (H.F.); 18339682437@163.com (J.L.); jfchen@fzu.edu.cn (J.C.); 2 Technical Innovation Service Platform for High-Value and High-Quality Utilization of Marine Organism, Fuzhou University, Fuzhou 350108, China; 3 Fujian Engineering and Technology Research Center for Comprehensive Utilization of Marine Products Waste, Fuzhou University, Fuzhou 350108, China; stephen6949@hit.edu.cn; 4 Fuzhou Industrial Technology Innovation Center for High-Value Utilization of Marine Products, Fuzhou University, Fuzhou 350108, China; 5 College of Life Sciences, Nanjing Agricultural University, Nanjing 210095, China; t2020061@njau.edu.cn; 6 State Key Laboratory of Urban Water Resource and Environment, School of Environment, Harbin Institute of Technology, Harbin 150090, China

**Keywords:** *Chlorella sorokiniana* FZU60, lycopene cyclase, α-carotene, β-carotene, lutein

## Abstract

Lutein is a high-value carotenoid with many human health benefits. Lycopene β- and ε-cyclases (LCYB and LCYE, respectively) catalyze the cyclization of lycopene into distinct downstream branches, one of which is the lutein biosynthesis pathway, via α-carotene. Hence, LCYB and LCYE are key enzymes in lutein biosynthesis. In this study, the coding genes of two lycopene cyclases (CsLCYB and CsLCYE) of a lutein-enriched marine green microalga, *Chlorella sorokiniana* FZU60, were isolated and identified. A sequence analysis and computational modeling of CsLCYB and CsLCYE were performed using bioinformatics to identify the key structural domains. Further, a phylogenetic analysis revealed that CsLCYB and CsLCYE were homogeneous to the proteins of other green microalgae. Subcellular localization tests in *Nicotiana benthamiana* showed that CsLCYB and CsLCYE localized in chloroplasts. A pigment complementation assay in *Escherichia coli* revealed that CsLCYB could efficiently β-cyclize both ends of lycopene to produce β-carotene. On the other hand, CsLCYE possessed a strong ε-monocyclase activity for the production of δ-carotene and a weak ε-bicyclic activity for the production of ε-carotene. In addition, CsLCYE was able to catalyze lycopene into β-monocyclic γ-carotene and ultimately produced α-carotene with a β-ring and an ε-ring via γ-carotene or δ-carotene. Moreover, the co-expression of CsLCYB and CsLCYE in *E. coli* revealed that α-carotene was a major product, which might lead to the production of a high level of lutein in *C. sorokiniana* FZU60. The findings provide a theoretical foundation for performing metabolic engineering to improve lutein biosynthesis and accumulation in *C. sorokiniana* FZU60.

## 1. Introduction

Lutein, as one of the primary xanthophylls, is predominantly found in vascular plants and green algae, where it plays crucial roles in facilitating photosynthesis and photoprotection [1]. Additionally, lutein can be obtained through dietary intake, providing various health benefits for human beings such as antioxidant and anti-inflammatory effects and immunological enhancement [2]. Currently, commercial lutein is mainly produced from marigolds, a system that is inadequate for meeting market demands due to its low production efficiency and high cost [3]. In recent years, microalgae have been considered promising new sources of lutein due to their rapid growth rate, high lutein content, and easy cultivation requirements [3].

In vascular plants and green algae, the biosynthesis of carotenoids initiates from isopentenyl diphosphate (IPP) and its isomeride dimethylallyl diphosphate (DMAPP), which are catalyzed into geranyl diphosphate (GGPP). Further, two molecules of GGPP are catalyzed into phytoene by phytoene synthase (PSY) [4]. Then, the phytoene is desaturated by phytoene desaturase (PDS) and ζ- carotene desaturase (ZDS), followed by isomerization via 1, 5-cis-ζ-carotene isomerase (ZISO) and carotenoid isomerase (CRTISO) to produce lycopene. Subsequently, the carotenogenic pathway undergoes its first bifurcation point, at which lycopene β-cyclase (LCYB) and lycopene ε-cyclase (LCYE) play crucial roles in regulating the distribution of carotenoids [5]. Typically, LCYB and LCYE catalyze the cyclization of lycopene to introduce β- and ε-ionone end groups, respectively, leading to the production of β-carotene via γ-carotene and α-carotene via δ-carotene [6]. The biosynthesis of lutein from α-carotene occurs due to the catalyzation of carotene hydroxylases. Consequently, the formation of α-carotene through the asymmetric ε- and β-cyclization of lycopene is identified as the pivotal rate-limiting step in lutein biosynthesis, which is primarily influenced by the internal competition between LCYB and LCYE [7].

Lycopene cyclases (LCYs) have been fully characterized in several species, including cyanobacteria (CrtLb and CrtLe) and plants and algae (LCYB and LCYE). According to their cyclization ability and catalytic properties, LCYs can be categorized as mono-cyclic or bi-cyclic and as mono-functional or bi-functional enzymes. LCYBs from most organisms exhibit strong levels of double β-ring activity and possess a mono-functional cyclizing ability, facilitating the transformation of lycopene into β-carotene. Generally, the γ-carotene intermediate is not detected due to the robust activity of LCYB [8]. Examples of such enzymes include the LCYBs from *Synechococcus elongatus* PCC 7942 [9] and *Prochlorococcus marinus* [10] in cyanobacteria, *Arabidopsis thaliana* [11] and *Zea mays* [12] in plants, and *Porphyra umbilicalis*, *Pyropia yezoensis* [13], and *Haematococcus pluvialis* [8] in algae. However, both γ-carotene and β-carotene were derived by LCYB from a red alga, *Bangia fuscopurpurea* [14]. In addition, the LCYB from *Dunaliella bardawil* (DbLCYB) is notable for its relatively weak lycopene ε-monocyclase activity except for the lycopene β-monocyclase and bicyclase activities that convert lycopene into γ-carotene and β-carotene [15]; it is the sole LCYB with bifunctional activity identified thus far. In most organisms, the LCYE is typically an ε-monocyclase which functions as a mono-functional cyclase to convert lycopene into δ-carotene, coupling with LCYB to form α-carotene. For example, the LCYEs of *H. pluvialis* [8] and *Chromochloris zofingiensis* [16] only exhibit ε-monocyclase activity and can solely catalyze the conversion of lycopene into δ-carotene. However, the LCYEs from *Lactuca sativa* [17], *Oryza sativa* [18], and *Osmanthus fragrans* [19] possess ε-bicyclase activity and can convert lycopene into ε-carotene. Additionally, LCYEs from some species also possess the β-monocyclase activity required to cyclize lycopene into γ-carotene in *Escherichia coli*, such as CrtLe from *Proch. marinus* MED4 [10] and DbLCYE from *D. bardawil* FACHB-847 [15]. Therefore, the relative enzymatic activities and functions of LCYB and LCYE may vary among different species, ultimately determining the carotenoid composition and content in each branch of the carotenogenic pathway.

*Chlorella sorokiniana* FZU60, a lutein-enriched green microalga isolated from coastal areas, can be cultured under various trophic conditions to achieve efficient lutein production [20,21,22]. However, the LCYs of *C. sorokiniana* FZU60 have not been characterized, hindering our understanding of the molecular mechanism of the high lutein-producing ability of this strain. In this study, gene cloning, bioinformatics analysis, and the subcellular localizations of lycopene β- and ε-cyclases from *C. sorokiniana* FZU60 (CsLCYB and CsLCYE) were conducted. Moreover, the functions of CsLCYB and CsLCYE were characterized via genetic complementation in *E. coli*. These results offer valuable insights into the biofunctions of CsLCYB and CsLCYE while establishing a foundation for fine-tuning lutein synthesis in *C. sorokiniana* FZU60, as well as other algae.

## 2. Results

### 2.1. Gene Cloning and Sequence Analysis of CsLCYB and CsLCYE

Based on the transcriptome data of *C. sorokiniana* FZU60, the full-length coding sequences (CDSs) of the *CsLCYB* and *CsLCYE* genes were 1629 bp and 1770 bp, respectively. The gene cloning and sequencing results indicated that the lengths and base sequences of the obtained CDSs of *CsLCYB* and *CsLCYE* genes were the same as those of the transcriptome data. The deduced full-length CsLCYB and CsLCYE proteins consisted of 542 and 589 amino acid residues, respectively (Table 1). The sequences of CsLCYB and CsLCYE have been submitted to GenBank under the accession numbers OR145861 and OR145862, respectively. A further analysis demonstrated that the molecular weight (MW) was 59,214.16 Da for CsLCYB and 62,916.23 Da for CsLCYE, the isoelectric point (pI) was 8.91 for CsLCYB and 6.78 for CsLCYE, the instability index was 42.58 for CsLCYB and 45.55 for CsLCYE, the aliphatic index (AI) was 88.78 for CsLCYB and 79.22 for CsLCYE, and the grand average of hydropathicity (GRAVY) was −0.063 for CsLCYB and −0.151 for CsLCYE (Table 1). Additionally, the online analysis carried out using the web software YLoc and Wolf PROST indicated that both CsLCYB and CsLCYE proteins were localized in chloroplasts (Table 1).

### 2.2. Structure Characterization of CsLCYB and CsLCYE

**Figure 1:**
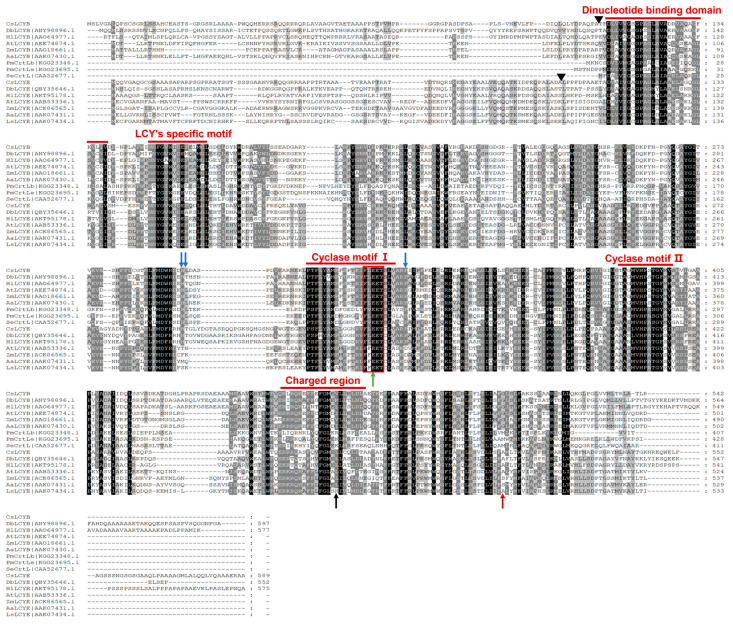
Alignment of the amino acid sequences of LCYBs and LCYEs. Aa—*Adonis aestivalis*; At—*Arabidopsis thaliana*; Cs—*Chlorella sorokiniana* FZU60; Db—*Dunaliella bardawil* FACHB-847; Hl—*Haematococcus lacustris*; Ls—*Lactuca sativa*; Pm—*Prochlorococcus marinus* str. SS35; Se—*Synechococcus elongatus* PCC 7942; Zm—*Zea Mays*. The black triangles indicate the proposed cleavage sites of the transit peptides of CsLCYB and CsLCYE. The amino acid residues indicated by the blue arrows are conserved in the LCYBs. The glutamate residue of the conserved FLEET motif indicated by the green arrow is supposed to be important for β-carotene formation. The histidine residue indicated by the black arrow is supposed to determine the ε-bicyclase function of LsLCYE. The serine indicated by the red arrow is supposed to determine the ε-bicyclase functions of ZmLCYE and AaLCYE.

The amino acid sequences of CsLCYB and CsLCYE were subjected to comparative analyses with those of other species. The results revealed that the amino acid sequences of the LCYs from cyanobacteria consisted of approximately 400 amino acids. In contrast, the LCYBs and LCYEs of the eukaryotic green algae (including CsLCYB and CsLCYE) and plants possessed an additional N-terminal region consisting of roughly 100 more amino acid residues than the cyanobacteria (Figure 1), which could be the N-terminal chloroplast transit peptide facilitating plastid localization [23]. The analysis that used the Pfam database revealed that the 112-520th amino acid residues of the CsLCYB protein and the 111-518th amino acid residues of the CsLCYE protein were in the typical LCY domain. Five highly conserved domains essential for catalytic activity, including the dinucleotide-binding domain, LCY-specific motif, cyclase motif Ⅰ, cyclase motif Ⅱ, and charged region [24,25], were identified in CsLCYB and CsLCYE (Figure 1). Similar to other LCYs, it was found that the dinucleotide binding domain, as well as cyclase motifs Ⅰ and Ⅱ of CsLCYB and CsLCYE, contained aromatic and carboxyl amino acid residues (such as phenylalanine, tyrosine, glutamic acid, and aspartic acid), which might represent negative point charges involved in coordinating incipient carotenoid carbocations [26].

**Figure 2:**
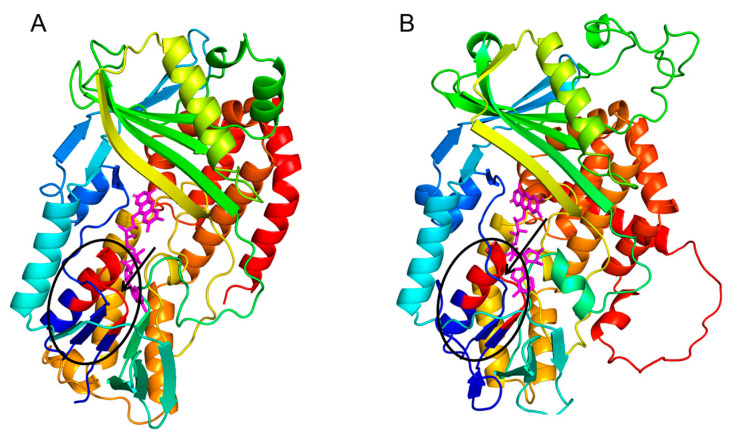
Predicted 3D structures of CsLCYB (**A**) and CsLCYE (**B**). The β chain-α helix-β chain responsible for binding FAD/NAD cofactor are indicated by a black ellipse, within which the V/IXGXGXXGXXXA motif is in red color. The FAD molecule is illustrated in a magenta color.

To elucidate the structural foundations of CsLCYB and CsLCYE, their 3D structures were predicted using the SWISS-MODEL server due to the lack of experimentally determined structures. The 3D structures of CsLCYB and CsLCYE were modified by eliminating the N-terminal chloroplast transit peptide since it is generally removed to generate a mature protein after plastid localization [11]. A Verify 3D analysis of CsLCYB and CsLCYE revealed that 83.87% and 86.18% of the amino acid residues scored above 0.2 in the 3D-1D profile, respectively (Figure S4), meeting the criterion of at least 80%. A Ramachandran plot analysis indicated that high percentages (93.5% and 91.6% for CsLCYB and CsLCYE, respectively) of residues were located within favored regions (Figure S4), thus confirming the reliability of the models [27]. In addition, the conserved secondary structure consisting of a β chain-α helix-β chain was apparent in both CsLCYB and CsLCYE (Figure 2 and Figure S5), which is consistent with other previously reported predicted models of LCYs [28,29].

### 2.3. Phylogenetic analysis of CsLCYB and CsLCYE

**Figure 3:**
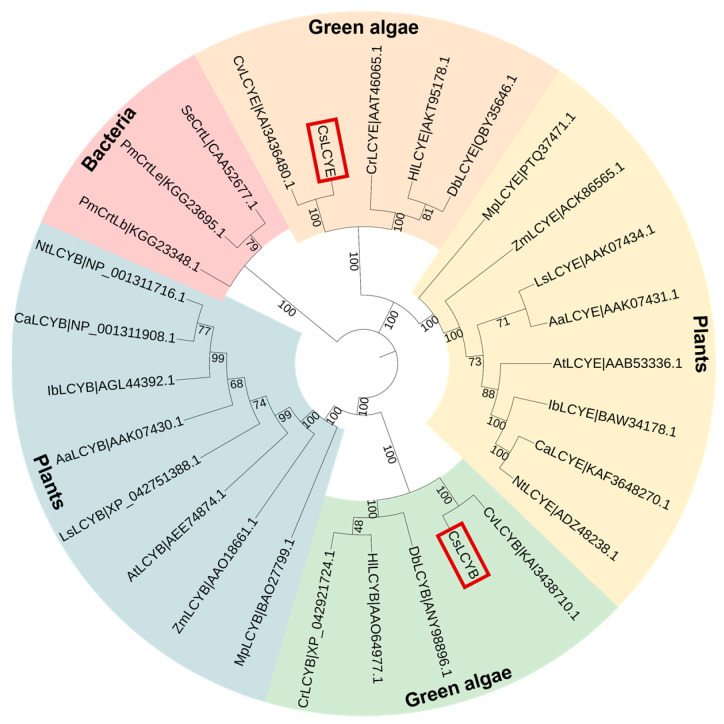
Cladogram of lycopene cyclases from green algae, plants, and cyanobacteria. The bootstrap values are indicated on each node. The number right after each protein is the GenBank ID. The CsLCYB and CsLCYE are highlighted in red boxes. Aa—*Adonis aestivalis*; At—*Arabidopsis thaliana*; Ca—*Capsicum annuum*; Cr—*Chlamydomonas reinhardtii*; Cs—*Chlorella sorokiniana*; Cv—*Chlorella vulgaris*; Db—*Dunaliella bardawil*; Hl—*Haematococcus lacustris*; Ib—*Ipomoea batatas*; Ls—*Lactuca sativa*; Mp—*Marchantia polymorpha*; Nt—*Nicotiana tabacum*; Pm—*Prochlorococcus marinus* str. SS35; Se—*Synechococcus elongatus* PCC 7942; Zm—*Zea Mays*.

The phylogenetic tree revealed that CsLCYB and CsLCYE initially fused with the clusters of *C. vulgaris*, followed by other green microalgae and then plants (Figure 3). Thus, CsLCYB and CsLCYE are closely related to LCYBs and LCYEs from other green algae and vascular plants and are distantly related to bacterial CrtL cyclase. These findings are consistent with the homologous matching of the amino acid sequences of LCYBs and LCYEs, indicating differential evolutionary rates for the LCYBs and LCYEs across different species (Figure 1).

### 2.4. Subcellular Localizations of CsLCYB and CsLCYE

**Figure 4:**
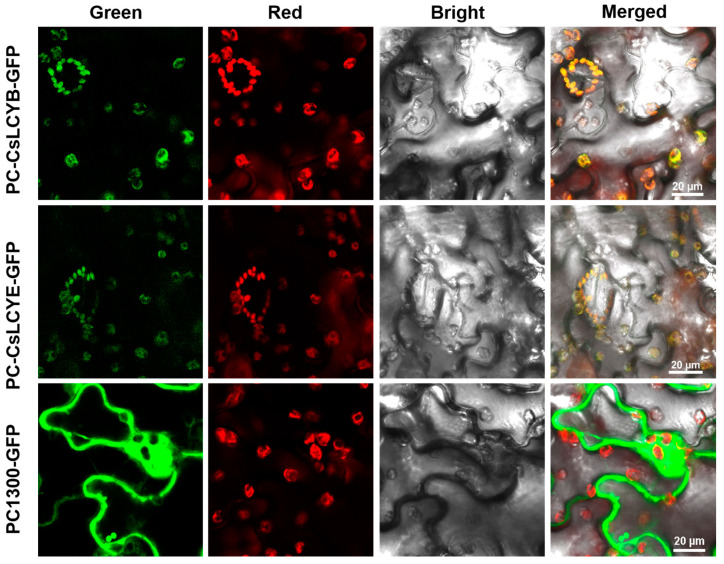
Subcellular localizations of CsLCYB and CsLCYE fused to GFP in *N. tabacum* leaf epidermal cells. Tobacco leaves that were only injected with GFP (PC1300-GFP) were used as a blank control. Tobacco leaves that were injected with CsLCYB and CsLCYE fused to GFP were indicated as PC-CsLCYB-GFP and PC-CsLCYE-GFP, respectively. The GFP fluorescence is shown in green. The chloroplast autofluorescence is shown in red. The bright field image was obtained under a light micrograph. The merged image shows the overlay of the abovementioned signals. The scale bar is 20 μm.

Understanding the subcellular localization of carotenoid biosynthesis enzymes is crucial for unveiling the mechanism of carotenoid biosynthesis. Therefore, it is valuable to perform localization assays of the LCY proteins. As shown in Figure 4, confocal scanning microscopic images demonstrate that the green fluorescence emitted due to the introduction of pCAMBIA1300-CsLCYB-GFP and pCAMBIA1300-CsLCYE-GFP was coincident with the red autofluorescence of the chloroplasts in *N*. *benthamiana* leaf epidermal cells, indicating their specific localization in chloroplasts.

### 2.5. Functional Identification of LCYs from C. sorokiniana FZU60

**Figure 5:**
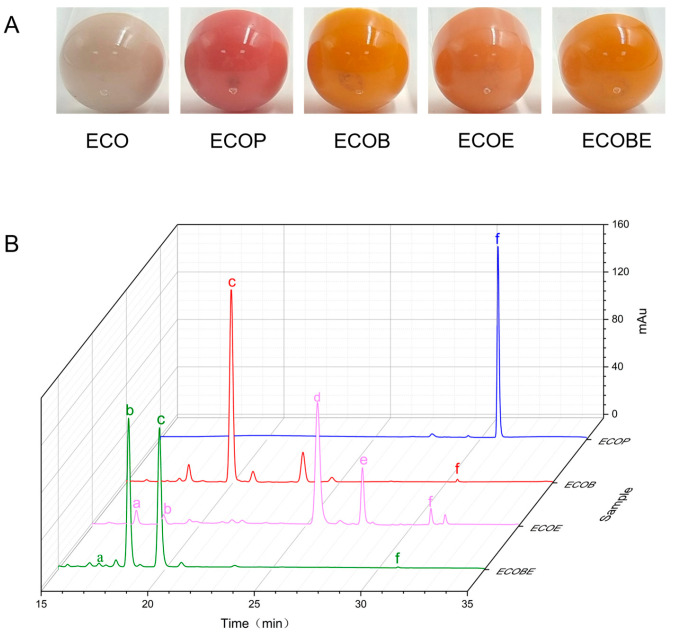
Identification of the functions of CsLCYB and CsLCYE in *E*. *coli*. (**A**) Colors of engineered *E. coli* strains; (**B**) carotenoid compositions of engineered *E. coli* strains. ECO—*E. coli* BL21(DE3); ECOP—*E. coli* BL21(DE3) carrying pAC-LYC and pTrc99a; ECOB—*E. coli* BL21(DE3) carrying pAC-LYC and pTrc-B; ECOE—*E. coli* BL21(DE3) carrying pAC-LYC and pTrc-E; ECOBE—*E. coli* BL21(DE3) carrying pAC-LYC and pTrc-BE; a—ε-carotene; b—α-carotene; c—β-carotene; d—δ-carotene; e—γ-carotene; f—lycopene.

The functional activity of the LCYs from *C. sorokiniana* FZU60 was assessed via genetic complementation with the lycopene-producing *E*. *coli* containing plasmid pAC-LYC. The results showed that the overexpression of CsLCYB and CsLCYE in red lycopene-producing *E*. *coli* changed the color of the bacteria into orange or orange-red (Figure 5A). As shown in Figure 5B, the introduction of CsLCYB into lycopene-producing *E. coli* (ECOB) resulted in a significant amount of β-carotene and a trace amount of uncyclized lycopene, indicating the robust lycopene β-cyclase activity of CsLCYB. After introducing CsLCYE, recombinant *E. coli* (ECOE) were found to produce a significant amount of δ-carotene, a moderate amount of γ-carotenoid, and small quantities of ε-carotene, α-carotene, and uncyclized lycopene. When CsLCYB and CsLCYE were co-introduced into lycopene-producing *E*. *coli* to construct ECOBE, α-carotene and β-carotene were mainly synthesized, with a slightly larger peak of α-carotene.

## 3. Discussion

Although LCYB and LCYE have been characterized from various plants, they have been only studied in a few green algae, including *H. pluvialis* [8], *D. salina* [30], *D. bardawil* [15], *C. zofingiensis* [16], *O. lucimarinus* [31], and *C. vulgaris* [29]. Due to the crucial roles of LCYs in lutein biosynthesis and the distinct functions of LCYs among different species, it is of great importance to characterize LCYB and LCYE from the lutein-enriched green microalga *C. sorokiniana* FZU60. The results of gene cloning and sequence analysis demonstrated that the lengths of CsLCYB and CsLCYE and their coding genes were similar to those of other green algae (Table 1 and Figure 1). Structure characterization revealed that CsLCYB and CsLCYE each had an additional N-terminal chloroplast transit peptide and highly conserved LCY domains, as seen in other green algae (Figure 1). In addition, the phylogenetic analysis indicated that CsLCYB and CsLCYE were grouped with the LCYBs and LCYEs of other green algae (Figure 3). Therefore, it is reasonable to confirm that the obtained sequences were the LCYB and LCYE of *C. sorokiniana* FZU60.

The existence of an N-terminal chloroplast transit peptide in CsLCYB and CsLCYE (Figure 1) was further verified through the chloroplast localization of these two proteins via subcellular localization experiments (Figure 4). The transit peptide can be removed to generate a mature protein after plastid localization [11]. The cleavage site of the transit peptide is supposed to be a semi-conserved consensus sequence of AXA↓X, in which the arrow indicates the cleavage location and X represents any amino acid [32]. Hence, the cleavage sites of CsLCYB and CsLCYE might be the 105th Alanine and 97th Alanine, respectively (indicated by the black triangles in Figure 1).

The predicted 3D structures of the CsLCYB and CsLCYE without N-terminal chloroplast transit peptides showed a conserved secondary structure of a β chain-α helix-β chain (Figure 2 and Figure S5) corresponding to a V/IXGXGXXGXXXA motif in the dinucleotide binding domain, with X representing any amino acid (Figure 1), which is responsible for binding the FAD/NAD cofactor in lycopene cyclization [28,33]. It was proposed that the first, second, and third glycines of the V/IXGXGXXGXXXA motif allow for a tight turn of the main chain from the β-strand into the loop, the close contact of the main chain with the pyrophosphate of the nucleotide, and the close packing of the helix with the β-stand, respectively [33]. The FAD/NAD binding location was deemed to be the interface between the alpha and beta subunits of the β chain-α helix-β chain in the LCY model [28].

**Figure 6:**
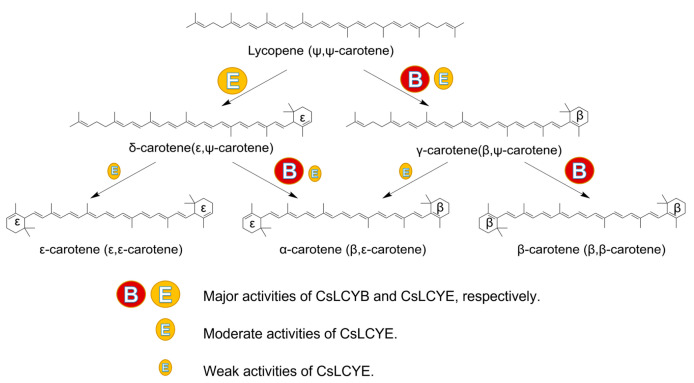
Probable pathway for lycopene cyclization in *C. sorokiniana* FZU60. The different circles of “B” and “E” denote the distinct activities of CsLCYB and CsLCYE.

The ECOB could produce β-carotene, but γ-carotene was not detected (Figure 5B), suggesting that CsLCYB possesses a strong double β-ring activity (Figure 6). The abovementioned findings are consistent with the functions of LCYBs from most plants and algae, such as *A*. *thaliana* [11], *Z. elegans* [34], *H. pluvialis* [8], *N. oceanica* [35], *Porph. umbilicalis*, and *P. yezoensis* [13]. The first glutamate (indicated by the green arrow in Figure 1) of a conserved FLEET motif in the cyclase motif I was supposed to be attributed to the formation of β-carotene [36]. In addition, consistent with a previous study [10], all β-ring cyclases, including CsLCYB, exhibited several conserved amino acid residues, including a histidine residue, a leucine or threonine residue, and a proline residue (indicated by the blue arrows in Figure 1), which might also be important for β-cyclase function. Except for β-cyclase function, the LCYBs of a few species have the ε-cyclase function, such as the LCYB from *D. bardawil* FACHB-847 [15]. However, the ε-cyclase function was not found for CsLCYB.

The ECOE could produce a significant amount of δ-carotene, a moderate amount of γ-carotenoid, and small quantities of ε-carotene and α-carotene (Figure 5B). The results suggest that CsLCYE possesses potent ε-monocyclase, weak ε-bicyclase, and moderate β-monocyclase activities (Figure 6). Although ε-carotene was produced in the ECOE, it was not detected in *C. sorokiniana* FZU60 (Figure S3). Similar results were found for the LCYEs from some plants, such as *L. sativa* [17], *O. sativa* [18], and *O. fragrans* [19], with ε-carotenoid being detected in a pigment complementation assay in *E. coli* but not in its native plants. These results could be due to the difference between the cytoplasmic environment of *E. coli* and the plastid environments of plants in terms of cofactors, membrane systems, and protein folding mechanisms, etc. [11,18,37]. One exception is the LCYE from *Lactuca sativa* (LsLCYE), which yields a substantial quantity of ε-carotene in the plant [17]. It has been found that the ε-bicyclase of LsLCYE is attributed to the special amino acid residue of histidine 457 [38], which was not found in other organisms (indicated by the black arrow in Figure 1). Regardless, it has been proposed that the S502 of ZmLCYE and the S494 of AaLCYE (indicated by the red arrow in Figure 1) might be responsible for the ε-bicyclase function [12]. However, the two kinds of the abovementioned amino acid residues were not found for CsLCYE, although it demonstrated ε-bicyclase activity. Thus, the specific structure responsible for ε-bicyclase function still needs to be explored further. On the other hand, CsLCYE possessed β-cyclase activity for catalyzing lycopene into γ-carotene, which was also found in PmCrtLe [10] and DbLCYE [15]. It is of note that α-carotene was produced by the ECOE, indicating that CsLCYE could further catalyze γ-carotene into α-carotene or δ-carotene into α-carotene. The production of α-carotene by LCYE is not commonly found in other organisms. To the best of our knowledge, it was only previously reported in ZmLCYE [12]. Thus, *C*. *sorokiniana* FZU60 may be able to produce more α-carotene at the bifurcation point of lycopene than most other organisms because it can catalyze lycopene into α-carotene via a single LCYE, with the exception of utilizing both LCYE and LCYB to produce α-carotene via δ-carotene.

α-carotene and β-carotene were mainly synthesized in the ECOBE (Figure 5B), indicating that CsLCYB also processes β-monocyclase activity for catalyzing δ-carotene into α-carotene (Figure 6) because α-carotene was only found in a small quantity when introducing a single CsLCYE. Moreover, the peak of α-carotene was larger than the peak of β-carotene in the ECOBE, which is consistent with the results reported for *D. bardawil* FACHB-847 [15] and *Marchantia polymorpha* [39]. In contrast, the co-introduction of LCYB and LCYE from *Porph. umbilicalis*, *P. yezoensis* [13], *B. fuscopurpurea* [14], and *Z. elegans* L. [34] into lycopene-producing *E*. *coli* resulted in a distinct product spectrum in which the level of β-carotene was significantly higher than the level of α-carotene. The production of a high level of α-carotene when co-introducing CsLCYB and CsLCYE might be due to biosynthesis via both δ-carotene and γ-carotene (Figure 6), which is not commonly found in other organisms. Since α-carotene is a crucial precursor for lutein biosynthesis, CsLCYB and CsLCYE’s ability to produce a high level of α-carotene might facilitate lutein accumulation, leading to the high lutein content found in *C*. *sorokiniana* FZU60.

## 4. Materials and Methods

### 4.1. Algae Strains and Culture Conditions

*C*. *sorokiniana* FZU60 was previously isolated from the coastal areas of Fujian Province, China [40]. To initiate a pre-culture, algal cells were transferred aseptically from an agar plate into a 1 L photobioreactor containing 1 L of BG11 medium. The pre-culture was then incubated under controlled conditions (33 °C, light intensity of 250 μmol/m^2^/s, and stir speed of 300 r/min) with a continuous supply of 0.15 vvm CO_2_ (2.5%) for approximately three days. For batch cultivation, the pre-cultured algae were harvested via centrifugation and inoculated at an initial concentration of 100 mg/L. All other cultural conditions remained unchanged from the pre-culture.

### 4.2. Gene Cloning of Lycopene Cyclases from C. sorokiniana FZU60

The RNA of *C. sorokiniana* FZU60 was extracted using a Biospin Plant Total RNA Extraction Kit (Bori, Hangzhou, China). It was then reversed into cDNA via a PrimeScript Total RT reagent Kit with a gDNA Eraser kit (TaKaRa, Shiga, Japan). According to the results of the de novo transcriptome sequencing of strain FZU60 [41], the CDSs of CsLCYB and CsLCYE were identified and used for primer design. The CDSs of the *CsLCYB* and *CsLCYE* genes were obtained via reverse transcription PCR (RT-PCR), using Phanta Max high-fidelity DNA polymerase (Vazyme, Nanjing, China). The PCR product was purified and inserted into the pET-28a vector using the ClonExpress^®^II one-step cloning kit (Vazyme, Nanjing, China). The *CsLCYB* and *CsLCYE* genes inserted into vector were sequenced by Shangya Biological Co., Ltd (Zhengzhou, China). The primers are shown in Table S2.

### 4.3. Bioinformatics Analysis

The CDSs of *CsLCYB* and *CsLCYE* genes were translated into protein sequence information via DNAMAN 9 software (Lynnon Biosoft, San Ramon, CA, USA). Then, the number of amino acids, MW, pI, instability index, AI, and GRAVY for each of the proteins were predicted by utilizing the ProtParam tool website (https://web.expasy.org/protparam/ (accessed on 10 March 2023)). The domains and functions of CsLCYB and CsLCYE were forecasted using the Interpro website (https://www.ebi.ac.uk/interpro/(accessed on 15 March 2023)), and their subcellular localizations were predicted via the YLoc website (https://abi-services.cs.uni-tuebingen.de/yloc/webloc.cgi (accessed on 18 March 2023)) and WoLFPSORT (https://wolfpsort.hgc.jp/ (accessed on 18 March 2023)) [42]. The secondary structures were predicted with the online software Phyre2 (http://www.sbg.bio.ic.ac.uk/phyre2/html/page.cgi?id=index (accessed on 20 March 2023)). The 3D structural models of the LCYB and LCYE were predicted using the SWISS-MODEL server (https://swissmodel.expasy.org/interactive (accessed on 10 May 2023)) and visualized with PyMOL software. The model quality was evaluated using a Ramachandran plot and Verify 3D via SAVES 6.0 (https://saves.mbi.ucla.edu/ (accessed on: 10 May 2023)). The CsLCYB-FAD and CsLCYE-FAD complexes were predicted via GalaxyWEB (https://galaxy.seoklab.org/cgi-bin/submit.cgi?type=DOCK (accessed on: 15 May 2023)). The LCYBs and LCYEs of green algae, plants, and bacteria were downloaded from GenBank. ClustalX2 software was used to perform multiple sequence alignment. A phylogenetic analysis of the amino acid sequences of different LCYs was carried out using MEGA X software with the neighbor-joining (NJ) method [43].

### 4.4. Subcellular Localizations of CsLCYB and CsLCYE

According to the analysis by YLoc (https://abi-services.cs.uni-tuebingen.de/yloc/webloc.cgi (accessed on 25 March 2023)) and WoLF PSORT (https://wolfpsort.hgc.jp/ (accessed on 25 March 2023)), the CsLCYB and CsLCYE proteins are likely located in chloroplasts. To verify this hypothesis, CsLCYB and CsLCYE, after removing the termination codon of the coding gene, were fused with a GFP protein at the N-terminal and integrated into the pCAMBIA1300 vector. The constructed plasmids pCAMBIA1300-CsLCYB-GFP, pCAMBIA1300-CsLCYE-GFP, and pCAMBIA1300-GFP (Table S1) were transformed into the *Agrobacterium tumefaciens* strain GV1301 for transient expression in the leaf epidermal cells of *Nicotiana benthamiana* via *Agrobacterium* infiltration [44]. A confocal laser microscope (Nikon, Tokyo, Japan) was used to observe the infiltrated tobacco leaves, with the GFP signal and chloroplast autofluorescence signal being captured by 488 nm and 640 nm excitation lasers, respectively [45].

### 4.5. Functional Identification of CsLCYB and CsLCYE in E. coli

The *E. coli* DH5 α strain was utilized as the host for plasmid proliferation, and *E. coli* BL21 (DE3) served as the expression host. The plasmids and strains employed for the functional identification of CsLCYB and CsLCYE are presented in Table S1. The lycopene-producing plasmid pAC-LYC, harboring *crtE*, *crtB*, and *crtI* genes from *Pantoea agglomerans* [13,46], was generously provided by Professor Shan Lu (Nanjing University, Nanjing, China). For vector construction, the ClonExpress^®^II one-step cloning kit (Vazyme, Nanjing, China) was utilized to ligate the PCR products of the *CsLCYB* and *CsLCYE* genes into the linearized pTrc99a vector, generating the plasmids pTrc-B and pTrc-E. In addition, the *CsLCYB* (with the termination codon removed) and *CsLCYE* genes were co-inserted into the pTrc99a vector to generate the plasmid pTrc-BE. Subsequently, the pAC-LYC plasmid was co-transformed with pTrc99a, pTrc-B, pTrc-E, and pTrc-BE into *E. coli* BL21 (DE3) to construct ECOP, ECOB, ECOE, and ECOBE. A single colony of *E*. *coli* was inoculated into 50 mL of Luria–Bertani (LB) medium supplemented with 30 μg/mL of kanamycin and 50 μg/mL of chloramphenicol, and then cultured overnight at 37 °C and 200 rpm. The bacterial culture (1 mL) was then inoculated into 100 mL of LB medium containing the same antibiotics and incubated at 37 °C and 200 rpm until the optical density (OD) at 600 nm reached a value of 0.8. Subsequently, protein expression was induced by adding 1.0 mM of isopropyl β-D-1thiogalactopyranoside (IPTG) and incubating the mixture for a further 48 h at 30 °C and 200 rpm.

### 4.6. Extraction and Determination of Carotenoids from E. coli

The aforementioned *E. coli* cultures were centrifuged at 5000× *g* for 5 min. The bacteria cells were collected and freeze-dried. The dried cells were mixed with 3 mL of acetone via vortexing and soaked for 2 h. The mixture was centrifuged at 5000× *g* for 5 min, and the pigment phase was collected. The pellet was extracted repeatedly until the solution became colorless. Subsequently, the collected pigment solution was dried with the use of nitrogen gas and dissolved in 1 mL of dichloromethane. The composition and content of carotenoids were determined via a high-performance liquid chromatograph (LC-16AT, Shimadzu, Kyoto, Japan) equipped with a photodiode array detector (PAD) and a reverse-phase C30 YMC carotenoid column (5 μm, 250 mm × 4.6 mm). The mobile phase consisted of an A phase, including 97% methanol (3% H_2_O) with the addition of 0.05 M of ammonium acetate and 0.1% butylated hydroxytoluene (BHT), and a B phase, including methyl tert-butyl ether (MTBE) containing 0.1% BHT (B phase). The column temperature was set at 25 °C. The injection volume was 10 μL. Before injection, all analytical pure reagents were filtered using a 0.22 μm microporous membrane. The gradient elution protocol was carried out according to the previous study [47]. Carotenoids detected within the range of 200–700 nm were identified by comparing them with carotenoid standards, according to their corresponding retention time and specific absorption spectra [47]. The standards for lycopene, δ-carotene, ε-carotene, γ-carotene, α-carotene, and β-carotene were procured from Sigma-Aldrich (St. Louis, MO, USA). The corresponding retention times and absorbance spectra of the carotenoids analyzed via HPLC are illustrated in Figures S1 and S2, respectively.

## 5. Conclusions

In this study, lycopene cyclases of a lutein-enriched green microalga *C. sorokiniana* FZU60 were identified, and their functions were characterized. The CsLCYB and CsLCYE had similar lengths and conserved domains, as well as relatively close evolutionary relationships with the LCYBs and LCYEs from other green microalgae, indicating that the obtained sequences were the LCYB and LCYE of *C. sorokiniana* FZU60. In addition, the CsLCYB and CsLCYE were localized in chloroplasts according to subcellular localization tests performed in *N. benthamiana*. Based on the genetic complementarity in *E. coli*, CsLCYB only has potent β-cyclase activity, while CsLCYE possesses potent ε-monocyclase, weak ε-bicyclase, and moderate β-monocyclase activities. Ultimately, α-carotene can be synthesized via two pathways, through δ-carotene or γ-carotene via catalyzation by CsLCYB and CsLCYE, which may result in a high level of α-carotene. Hence, *C. sorokiniana* FZU60 can obtain a large amount of α-carotene to synthesize lutein, leading to high levels of lutein in microalgal cells.

## Figures and Tables

**Table 1:** Sequence information for CsLCYB and CsLCYE.

Unigene	ORF Length (bp)	Protein Length (aa)	MW (Da)	pI	Instability Index	AI	GRAVY	Subcellular Localization
CsLCYB	1629	542	59,214.16	8.91	42.58	88.78	−0.063	Chloroplast
CsLCYE	1770	589	62,916.23	6.78	45.55	79.22	−0.151	Chloroplast

## Data Availability

The data presented in this study are available upon request from the corresponding author.

## Supplementary Materials

The following supporting information can be downloaded at: https://www.mdpi.com/article/10.3390/md21070418/s1, Figure S1: The corresponding retention times of carotenoids analyzed via HPLC; Figure S2: Absorbance spectra of carotenoids analyzed via HPLC; Figure S3: Carotenoid analysis of *C. sorokiniana* FZU60 via HPLC; Figure S4: Evaluation and validation of the tertiary structure models of CsLCYB and CsLCYE; Figure S5: Predicted structures of CsLCYB and CsLCYE; Table S1: Plasmids and strains used in this study; Table S2: Homologous sequences of plasmids and primer sequences for cloning *CsLCYB* and *CsLCYE* genes.
