# Supplementary material for: Functional Characterization of Lycopene β- and ε-Cyclases from a Lutein-Enriched Green Microalga Chlorella sorokiniana FZU60
Source: Mar Drugs. 2023 Jul 23;21(7):418. doi: 10.3390/md21070418 (PMC10381880; doi:10.3390/md21070418)
Supplement: Supplementary file 1 [file marinedrugs-21-00418-s001.zip › marinedrugs-2505004-supplementary.pdf]

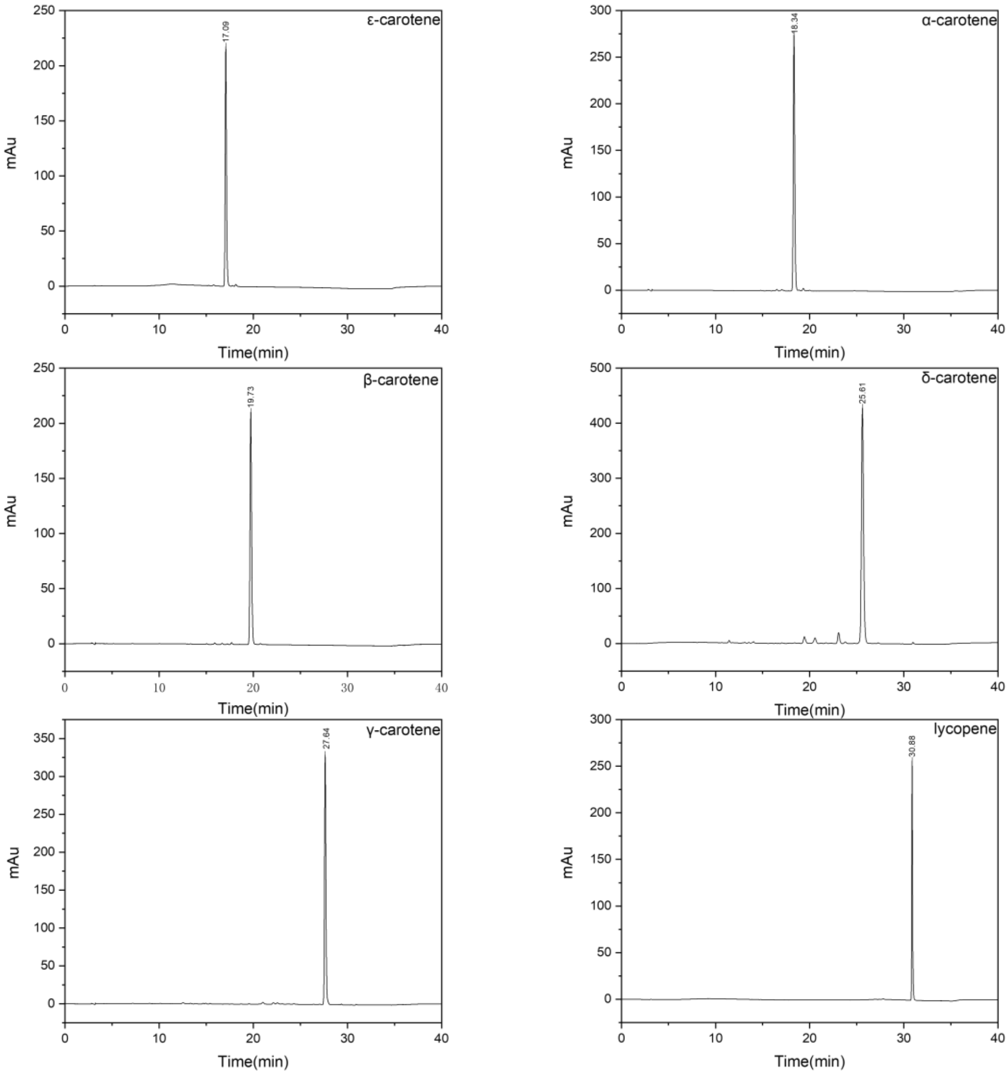

Figure S1 The corresponding retention time of carotenoids analyzed by HPLC.

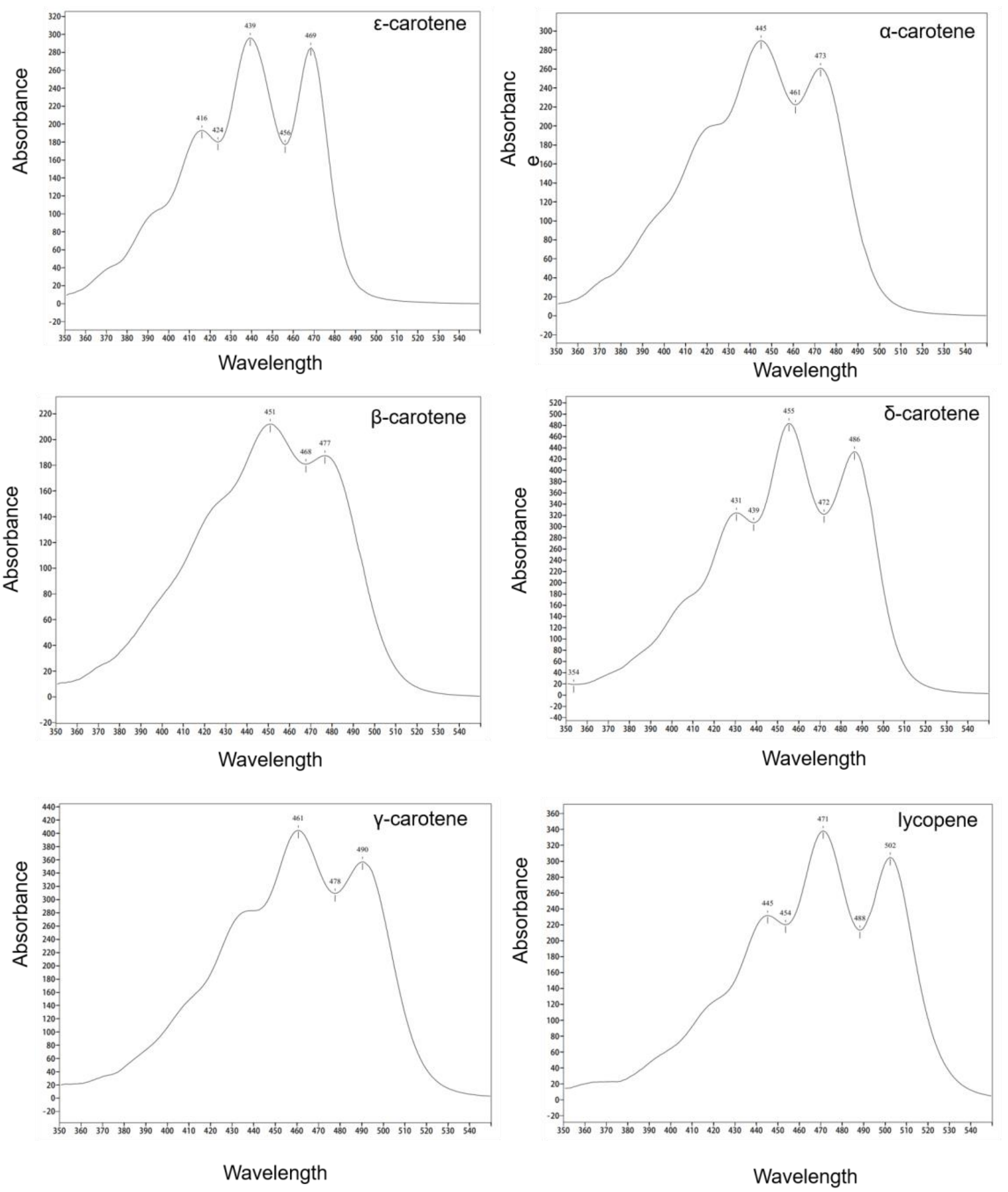

Figure S2 Absorbance spectra of carotenoids analyzed by HPLC.

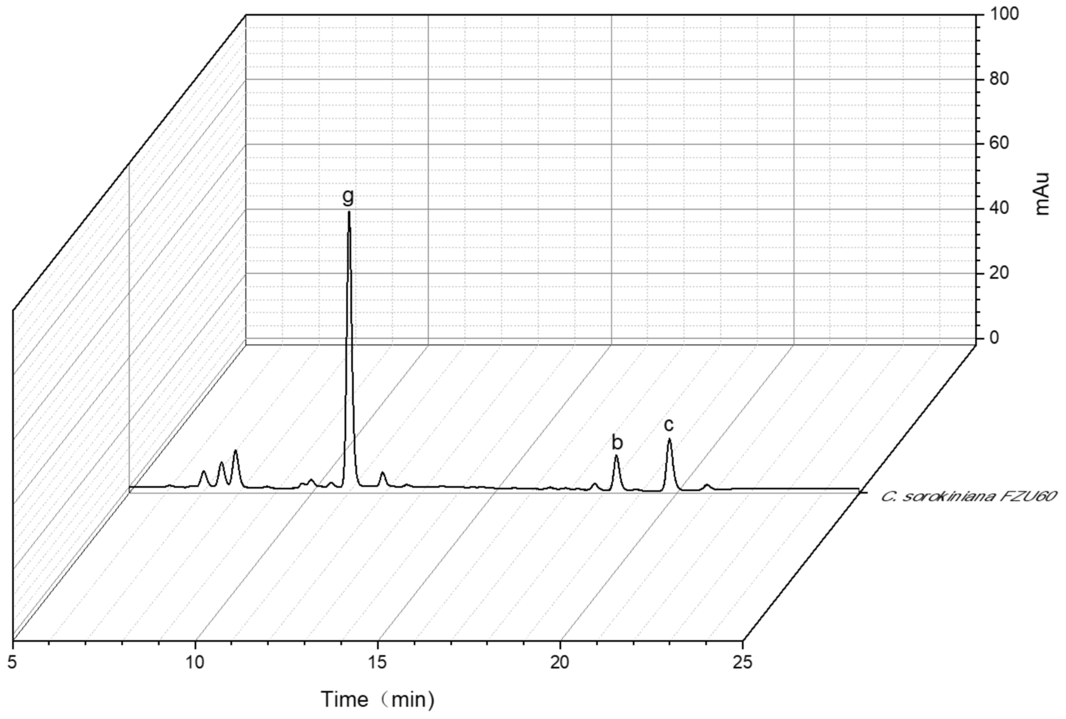

Figure S3 Carotenoid analysis of *C. sorokiniana* FZU60 by HPLC. Peak b, α-carotene; c, β-carotene; g, Lutein.

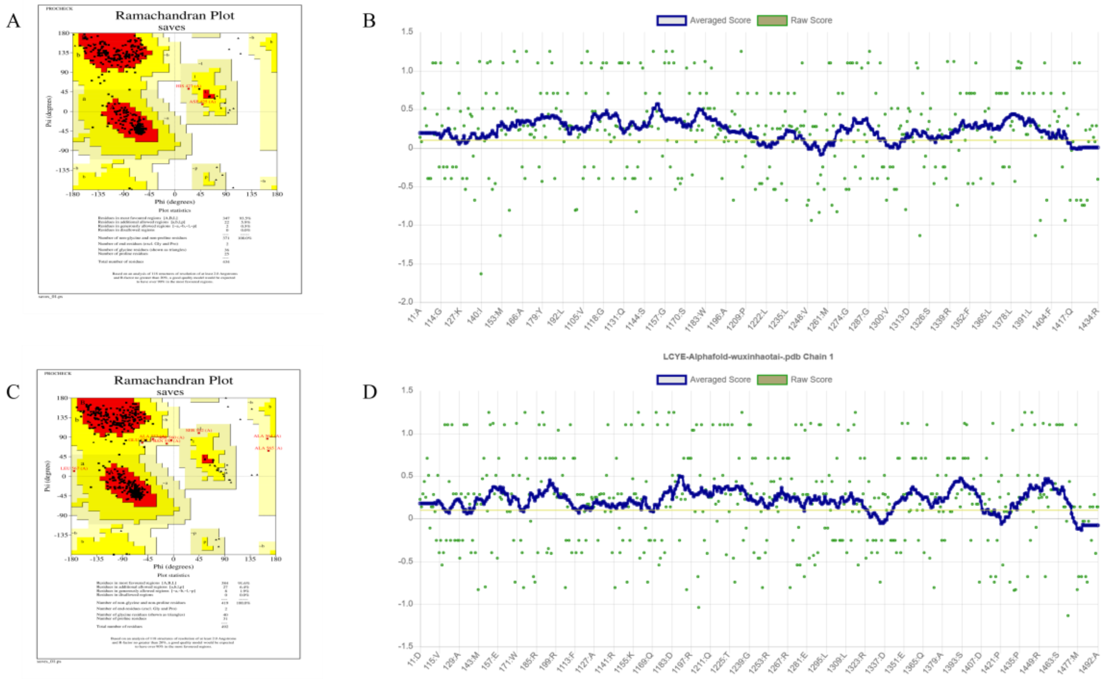

Figure S4 Evaluation and validation of the tertiary structure model of CsLCYB and CsLCYE. A, Ramachandran plot of CsLCYB; B, Verify 3D analysis result of CsLCYB; C, Ramachandran plot of CsLCYE; D, Verify 3D analysis result of CsLCYB.

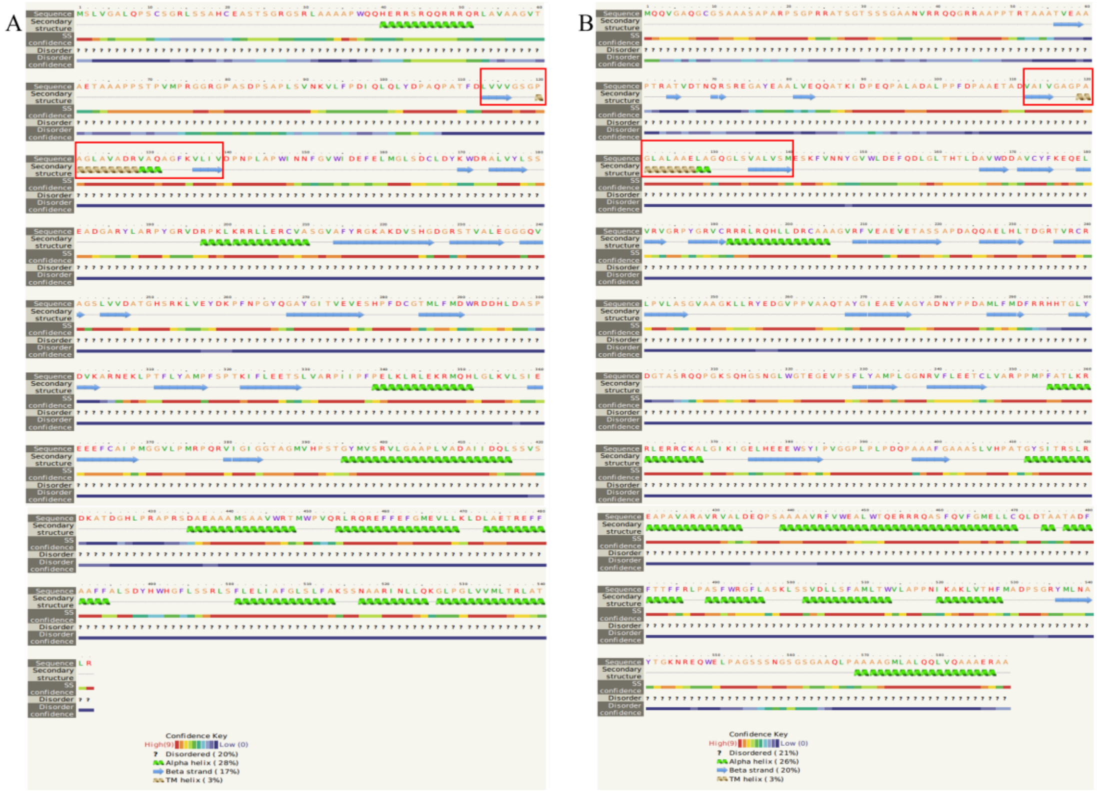

Figure S5 Predicted structures of CsLCYB (A) and CsLCYE (B). The conserved secondary structure of β chain-α helix-β chain for binding FAD/NAD cofactor is indicated by red box.

Table S1 Plasmids and strains used in this study.

Name	Characteristics
**Plasmids**	
pET-28a	Expression vector
pTrc99a	Expression vector
pCAMBIA1300	Expression vector
pAC-LYC	pACYCDuet-1 carrying *crtE*, *crtI*, and *crtB* genes from *Pantoea agglomerans*
pET-B	pET -28a carrying *CsLCYB* gene (*Bam*H I)
pET-E	pET -28a carrying *CsLCYE* gene (*Bam*H I)
pTrc-B	pTrc99a carrying *CsLCYB* gene (*Eco*R I and *Hin*d III)
pTrc-E	pTrc99a carrying *CsLCYE* gene (*Eco*R I and *Hin*d III)
pTrc-BE	pTrc99a carrying *CsLCYB* and *CsLCYE* genes (*Eco*R I and H*in*d III)
pCAMBIA1300-CsLCYB-GFP	pCAMBIA1300 carrying *CsLCYB* and *GFP* genes (*Kpn* I and *Pst* I)
pCAMBIA1300-CsLCYE-GFP	pCAMBIA1300 carrying *CsLCYE* and *GFP* genes (*Kpn* I and *Pst* I)
**Strains**	
E. coli DH5α	Host for proliferation
E. coli BL21(DE3)	Host for expression
ECOP	*E. coli* BL21(DE3) carrying pAC-LYC and pTrc99a
ECOB	*E. coli* BL21(DE3) carrying pAC-LYC and pTrc -B
ECOE	*E. coli* BL21(DE3) carrying pAC-LYC and pTrc -E
ECOBE	*E. coli* BL21(DE3) carrying pAC-LYC and pTrc-BE
Atu-B	*Agrobacterium tumefaciens* strain GV1301 carrying pCAMBIA1300-CsLCYB-GFP
Atu-E	*Agrobacterium tumefaciens* strain GV1301 carrying pCAMBIA1300-CsLCYB-GFP

Table S2 Homologous sequences of plasmids and primer sequences for cloning *CsLCYB* and *CsLCYE* genes.

Name	Primer		Amplicon length (bp)
**Plasmids**	**Homologous sequences (5'→3')**		
pET-28a	For	GGACAGCAAATGGGTCGCGGATCC	——
	Rev	GACGGAGCTCGAATTCGGATCC	
pTrc99a	For	AGGAAACAGACCATGGAATTC	——
	Rev	TCCGCCAAAACAGCCAAGCTT	
pCAMBIA1300	For	CGGGGGACGAGCTCGGTACC	——
	Rev	CCCTTGCTCACCATGGTACC	
**Gene**	**Forward and reverse primers (5'→3')**		
CsLCYB (for constructing pET-B and pTrc-B)	For	ATGTCGCTGGTCGGCGC	1629
	Rev	TCACCGCAGCGTGGCGA	
CsLCYE (for constructing pET-E and pTrc-E)	For	ATGCAGCAGGTGGGAGCCCA	1770
	Rev	TCACGCGGCCCGCTCT	
CsLCYB (for constructing pTrc-BE)	For	ATGTCGCTGGTCGGCGC	1626
	Rev	ACCTGCTGCATCCGCAGCGTGGCGAGCCGCGTCA	
CsLCYE (for constructing pTrc-BE)	For	ACGCTGCGGATGCAGCAGGTGGGAGCCCA	1770
	Rev	TCACGCGGCCCGCTCTGC	
CsLCYB (for constructing pCAMBIA1300-CsLCYB-GFP)	For	ATGTCGCTGGTCGGCGC	1626
	Rev	CCGCAGCGTGGCGAGCC	
CsLCYE (for constructing pCAMBIA1300-CsLCYB-GFP)	For	ATGCAGCAGGTGGGAGCCCA	1767
	Rev	CGCGGCCCGCTCTGCC	
